# Roughness of retinal layers in Alzheimer’s disease

**DOI:** 10.1038/s41598-021-91097-3

**Published:** 2021-06-03

**Authors:** Lucía Jáñez-García, Omar Bachtoula, Elena Salobrar-García, Rosa de Hoz, Ana I. Ramirez, Pedro Gil, José M. Ramirez, Luis Jáñez-Escalada

**Affiliations:** 1 Instituto de Investigaciones Oftalmológicas Ramón Castroviejo, Universidad Complutense de Madrid, Madrid, Spain; 2 Instituto de Tecnología del Conocimiento, Universidad Complutense de Madrid, Madrid, Spain; 3 Departamento de Inmunología, Oftalmología y ORL, Facultad de Óptica y Optometría, UCM, IdiSSC, Madrid, Spain; 4 Unidad de Memoria, Servicio de Geriatría, Hospital Clínico San Carlos, Madrid, Spain; 5 Departamento de Medicina, Facultad de Medicina, Universidad Complutense de Madrid, Madrid, Spain; 6 Departamento de Inmunología, Oftalmología y ORL, Facultad de Medicina (UCM), IdiSSC, Madrid, Spain; 7 Departamento de Psicobiología y Metodología en Ciencias del Comportamiento, Universidad Complutense de Madrid, Madrid, Spain

**Keywords:** Biomarkers, Neuroscience, Alzheimer's disease, Retina, Imaging techniques, Mathematics and computing

## Abstract

There is growing evidence that thinned retinal regions are interspersed with thickened regions in all retinal layers of patients with Alzheimer’s disease (AD), causing roughness to appear on layer thickness maps. The hypothesis is that roughness of retinal layers, assessed by the fractal dimension (FD) of their thickness maps, is an early biomarker of AD. Ten retinal layers have been studied in macular volumes of optical coherence tomography from 24 healthy volunteers and 19 patients with mild AD (Mini-Mental State Examination 23.42 ± 3.11). Results show that FD of retinal layers is greater in the AD group, the differences being statistically significant (p < 0.05). Correlation of layer FD with cognitive score, visual acuity and age reach statistical significance at 7 layers. Nearly all (44 out of 45) FD correlations among layers are positive and half of them reached statistical significance (p < 0.05). Factor analysis unveiled two independent factors identified as the dysregulation of the choroidal vascular network and the retinal inflammatory process. Conclusions: surface roughness is a holistic feature of retinal layers that can be assessed by the FD of their thickness maps and it is an early biomarker of AD.

## Introduction

Currently, one of the most frequent neurodegenerative diseases in the elderly is Alzheimer’s disease (AD). This pathology generates a serious problem in public health services in all developed and developing countries. It is therefore of the utmost importance to find biomarkers for this disease that enable the diagnosis in the earliest stages of the disease, support preventive measures and facilitate the development of new treatments. As shown in previous studies, AD patients experience visual system abnormalities, even in the earliest stages of the disease (visual acuity, contrast sensitivity, color vision and visual integration)^1–6^. The retina can be studied non-invasively, and retinal changes in patients with AD have been observed and are dependent on the progression of the AD^6–11^. Recent studies of the vascular ocular system using optical coherence tomography (OCT) and OCT-angiography have shown that, during the initial stages of AD, changes appear in the choroid, which are dependent on the ciliary vascular system, while the retinal system is preserved^12^. OCT is currently the most powerful imaging tool to examine the retina and its integrating layers; it has been widely used and has consistently shown significant changes mainly in the ganglion cell, nerve fiber and inner plexiform layers^6,13,14^; when combined with image processing techniques, a greater detail can be obtained about the structural changes caused by AD on retinal layers.

Growing evidence has shown that, even in the early stages of AD, thinned and thickened regions coexist in most retinal layers^6,15,16^. Interspersed thinned and thickened regions of differing sizes within the same layer increase the *topographical complexity* of their delimiting surfaces. Topographical complexity can be defined as the three-dimensional arrangement of structural features over the surfaces of retinal layers, spanning all spatial scales, and can be estimated by rugosity and roughness. *Rugosity* is an estimate of the topographic complexity based on a single measurement scale, while *roughness* is an estimate of the topographic complexity based on measures spanning a range of spatial scales (the definitions are adapted from Zawada^17^). We focused on retinal roughness (instead of rugosity) because thinned and thickened regions have different sizes and are interspersed in a variety of patterns, thereby affecting the topographical complexity of the layer’s surfaces in multiple spatial scales.

The roughness of a retinal layer can be assessed on its two delimiting surfaces; however, we decided to assess the roughness of its thickness map by computing its fractal dimension (FD): the greater the roughness of the surface, the greater its FD. We therefore expected higher FD values of the AD group in the layers studied: nerve fiber (NFL), ganglion cell (GCL), inner plexiform (IPL), inner nuclear (INL), outer plexiform (OPL), outer nuclear (ONL), inner segment/outer segment (IS/OS), outer segment (OSL), outer segment PR/RPE complex (OPR) and retinal pigment epithelium (RPE). Then we hypothesized that the roughness of the thickness maps of the retinal layers, as assessed by their FD, could provide valuable information on the presence of AD. The objective of the present study became twofold: (1) to investigate the feasibility of using FD to quantify the roughness of retinal layers and (2) to determine the usefulness of FD for the early detection of AD.

## Results

### Sample data

#### Demographic and clinical data

Table 1 shows the data for the patients with mild AD and the age-matched controls. The two groups showed no statistically significant differences in age, sex distribution, educational level or refractive error. The distribution of refractive error for the 2 groups was similar, and the difference was not statistically significant (Kolmogorov–Smirnov Z = 0.76; p = 0.60). The educational level was categorized in terms of years of education as 1 (< 9 years), 2 (9–17 years) or 3 (> 17 years). All patients scored higher than 17 on MMSE and the two groups showed a statistically significant difference in mean score.

**Table 1:** Demographic and clinical data of AD patients and controls.

	Alzheimer’s disease group	Control group	p-value
n	19	24	
Age^a^	79.16 ± 3.93	75.71 ± 2.83	0.59^c^
Mini-Mental State	23.42 ± 3.11	28.38 ± 2.02	** < 0.001**^c^
Examination^a^	Range = [17, 29]	Range = [25, 31]	
Educational level^b^	1 ± 1	1 ± 0	0.57^c^
**Sex**			0.90^d^
Male	6	8	
Female	13	16	
Race	Caucasian	Caucasian	
Refractive error^a^	0.39 ± 1.40	− 0.12 ± 1.13	0.18^e^
	Range = [− 2.00, 4.00]	Range = [− 2.75, 2.25]	

^a^Mean value ± SD.

^b^Median ± interquartile range.

^c^Mann–Whitney two-sided U-test.

^d^Proportions two-sided z-test.

^e^Student’s two-sided t-test.

In boldface p-value < 0.05 (SD, standard deviation).

#### Layer thickness data

**Figure 1:**
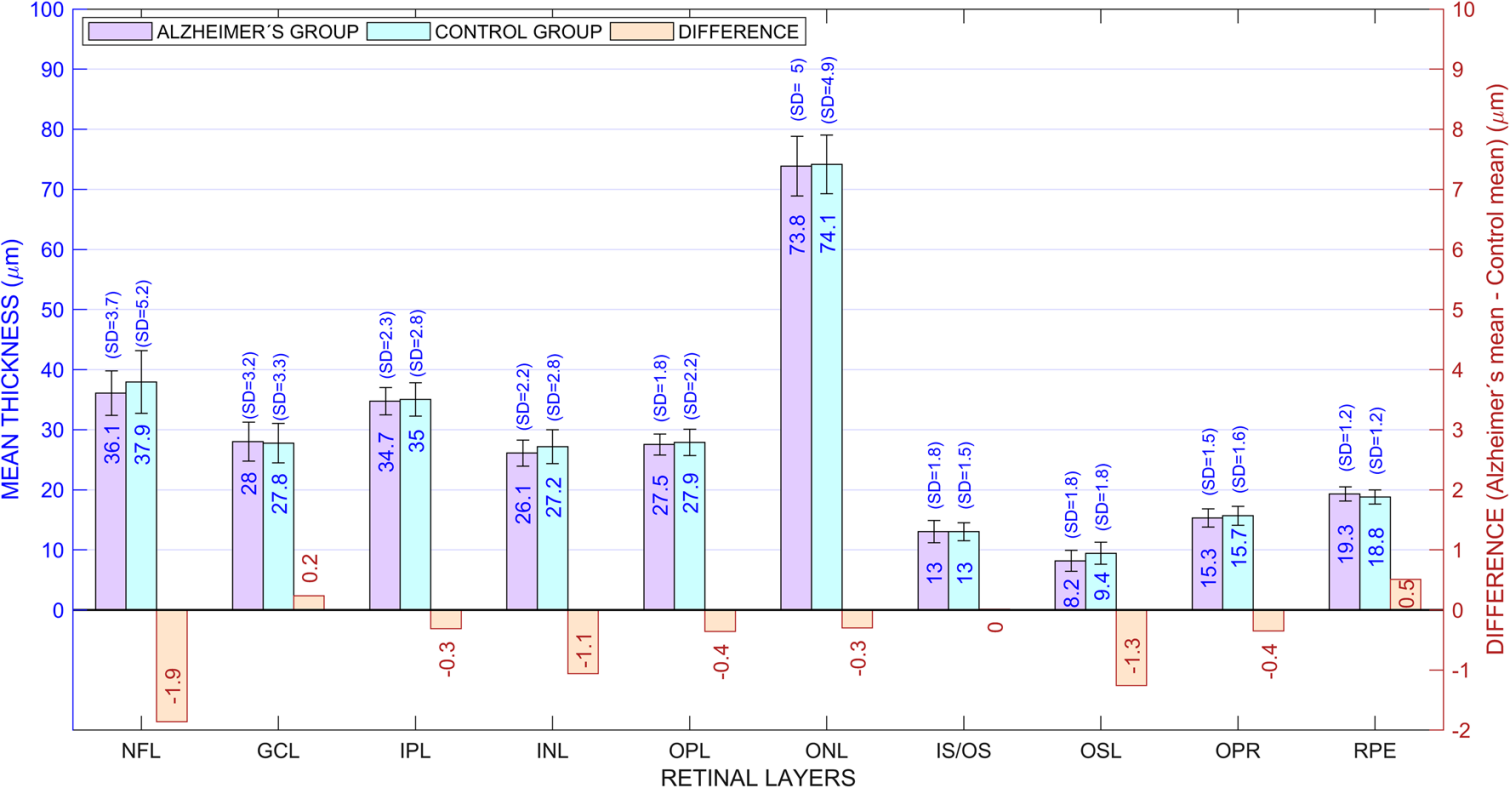
Thickness of retinal layers for patients and controls. The mean thickness is shown within the bars and the standard deviation (SD) is shown above. The difference between the two groups is displayed in orange bars, whose length has been amplified (× 10) to show the trend of retinal layers to be thinner in the AD group. *NFL* nerve fiber layer, *GCL* ganglion cell layer, *IPL* inner plexiform layer, *INL* inner nuclear layer, *OPL* outer plexiform layer, *ONL* outer nuclear layer, *IS/OS* inner segment/outer segment layer, *OSL* outer segment layer, *OPR* outer segment PR/RPE complex, *RPE* retinal pigment epithelium.

Figure 1 shows the mean retinal thickness of all retinal layers for each group. Although the mean retinal thickness was 5.1 µm thinner in the AD group than in the control group (mean ± standard deviation: 281.4 ± 11.9 µm and 286.5 ± 16.9 µm, respectively), the difference was not statistically significant (Student’s t = −1.08; df = 41; p = 0.14). Most of the retinal layers were slightly thinner in the AD group, although the difference was statistically significant only in the OSL (Student’s t = −2.23; df = 41; p = 0.02). Conversely, the patients had thicker RPE, but the difference did not reach statistical significance.

### Boundary surfaces and thickness maps

**Figure 2:**
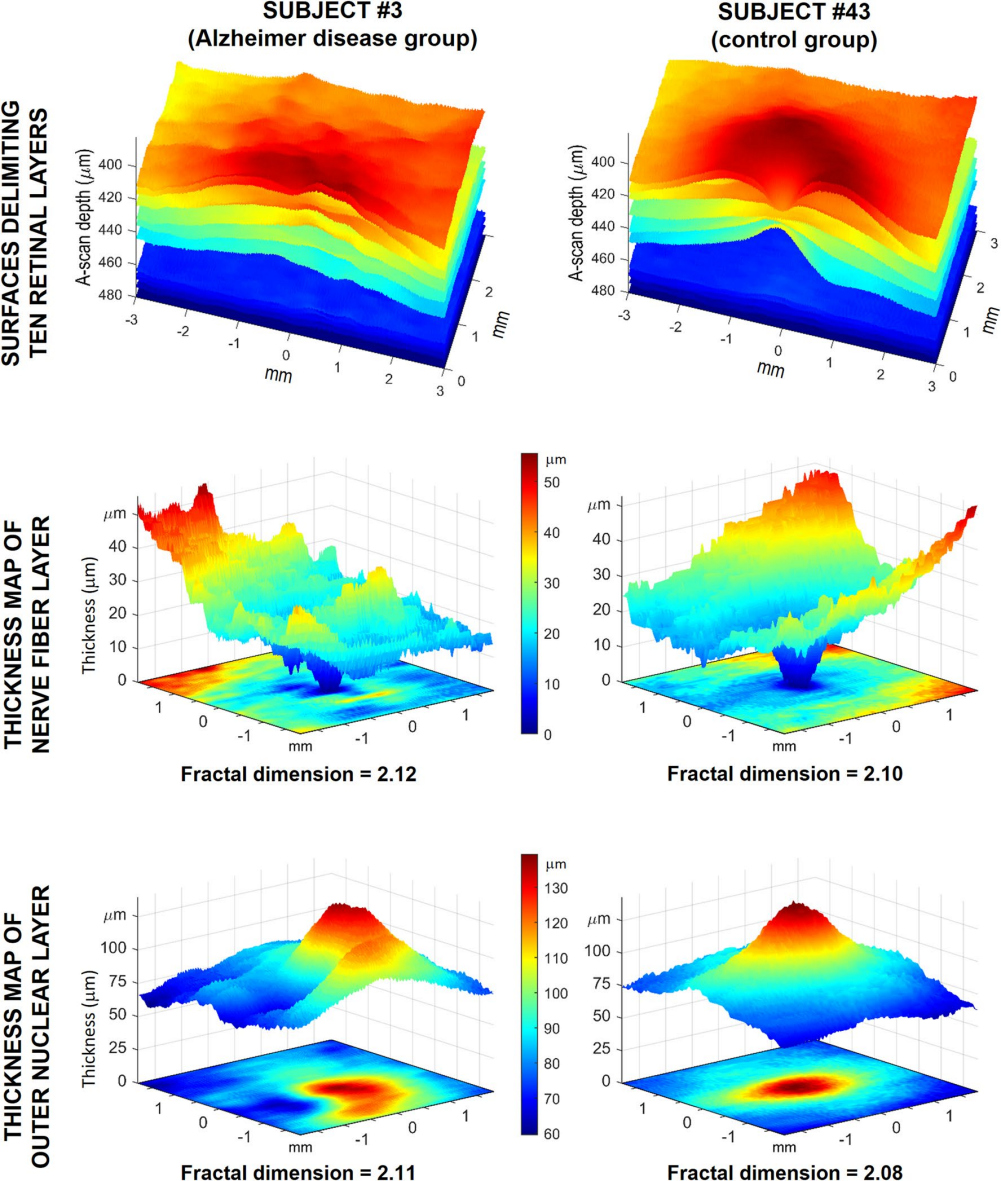
Roughness and fractal dimension of the retinal layers. Left column corresponds to the retina of a patient with Alzheimer’s disease (AD) (subject #3). The right column corresponds to the retina of a healthy participant (subject #43). The upper row shows the 11 surfaces delimiting the 10 retinal layers of both participants, clearly showing the greater roughness of the delimiting surfaces for the patient with AD. The middle and bottom rows show the thickness maps of the nerve fiber layer and the outer nuclear layers for the two subjects. Each thickness map is represented in two similar manners in the same figure: as a color-coded image at the foot of each figure and as a relief surface (or terrain elevation map) above. In the color-coded images, the roughness is visually demonstrated as texture or granularity. In the relief representations, the roughness is expressed by surface irregularities. Both representations lead to the conclusion that the roughness was greater for the patient with AD. The fractal dimension of each thickness map is a real number (slightly greater than 2) whose value reflects the roughness of each thickness map. Its higher value in both layers of the patient with AD indicates that their roughness is greater than that of the control participant, which agrees with the visual information. Image created using MATLAB (2018a) www.mathworks.com/products/matlab.

We obtained 11 surfaces delimiting 10 retinal layers for each participant and derived a thickness map for each layer, as described in the Methods section. For illustrative purposes, Fig. 2 shows the delimiting surfaces and thickness maps of 2 participants. The upper row shows the 11 delimiting surfaces and the corresponding 10 layers for a patient with AD in the left column and for a healthy participant in the right column; the medium and bottom rows show the thickness maps for their NFL and ONL, respectively. The greater roughness of delimiting surfaces and thickness maps becomes apparent in the surfaces on the left column, corresponding to the patient with AD.

### Roughness of the thickness maps of retinal layers

The roughness of the thickness maps changes among the various layers of a single retina and among participants, as shown in the two retinal layers of two participants in Fig. 2: the greater roughness is visually apparent in the left column, which corresponds to the patient with AD.

We calculated the FD of the thickness map as the roughness index for each retinal layer for all participants. The mean computing time for all layers of a single participant in our dedicated server took 1.2 s (standard deviation = 0.3). We obtained similar values when running the same MATLAB program on a typical laptop, thus suggesting that the FD can be computed even faster in clinical applications by using a compiled and optimized program.

#### Roughness in patients with AD versus control participants

Once we calculated the mean FD of the 10 layers for a participant, we obtained a set of 43 independent observations. By comparing the series of 19 patient values with the series of 24 control values, we found that the mean FD was greater in the patients, a difference that was statistically significant (2.1146 ± 0.0054 and 2.1122 ± 0.0043; Mann–Whitney U rank sum exact test W = 494, p = 0.03). When we performed the same comparison for the two groups using the mean FD of just the 9 neuronal layers (excluding the pigment epithelium), the results once again led to the conclusion that the mean FD was higher in the patients, a difference that was statistically significant (2.1133 ± 0.0056 for the patients and 2.1110 ± 0.0044 for the controls; Mann–Whitney U rank sum exact test W = 486, p = 0.049). The two results therefore lead to the conclusion that AD has the statistically significant effect of increasing the mean FD of retinal layers.

#### Roughness of the diseased layers versus control layers

Table 2 shows the mean (± standard deviation) roughness of the thickness map for each retinal layer for the AD and control groups.

**Table 2:** Fractal dimension of the thickness maps of retinal layers in Alzheimer disease patients and control groups.

Retinal layer	AD group FD mean ± SD	Control group FD mean ± SD	FD difference (AD – CTL) × 10^3^	Mann–Whitney	
				*U*	*p *value
**1. NFL**	2.1074 ± 0.0057	2.1018 ± 0.0040	**5.6**	552	0.000
2. GCL	2.1033 ± 0.0083	2.1012 ± 0.0061	2.1	458	0.168
3. IPL	2.1136 ± 0.0069	2.1110 ± 0.0060	2.6	472	0.096
4. INL	2.1105 ± 0.0080	2.1078 ± 0.0067	2.8	470	0.105
5. OPL	2.1180 ± 0.0076	2.1169 ± 0.0084	1.1	432	0.372
**6. ONL**	2.0939 ± 0.0088	2.0885 ± 0.0087	**5.3**	491	0.038
7. IS/OS	2.1281 ± 0.0081	2.1278 ± 0.0067	0.3	419	0.495
8. OSL	2.1217 ± 0.0103	2.1219 ± 0.0075	− 0.2	443	0.276
9. OPR	2.1230 ± 0.0106	2.1219 ± 0.0053	1.1	474	0.088
**10. RPE**	2.1269 ± 0.0071	2.1232 ± 0.0043	**3.7**	499	0.024
Total Retina	2.0782 ± 0.0105	2.0800 ± 0.0085	-1.7	384	0.799

Statistically significant differences (p < 0.05) and the corresponding layer names appear in boldface.

*AD* Alzheimer’s disease, *FD* fractal dimension, *SD*  standard deviation, *CTL* control (group), *NFL* nerve fiber layer, *GCL*  ganglion cell layer, *IPL* inner plexiform layer, *INL* inner nuclear layer, *OPL* outer plexiform layer, *ONL* outer nuclear layer, *IS/OS* inner segment/outer segment layer, *OSL* outer segment layer, *OPR* outer segment PR/RPE complex, *RPE* retinal pigment epithelium.

**Figure 3:**
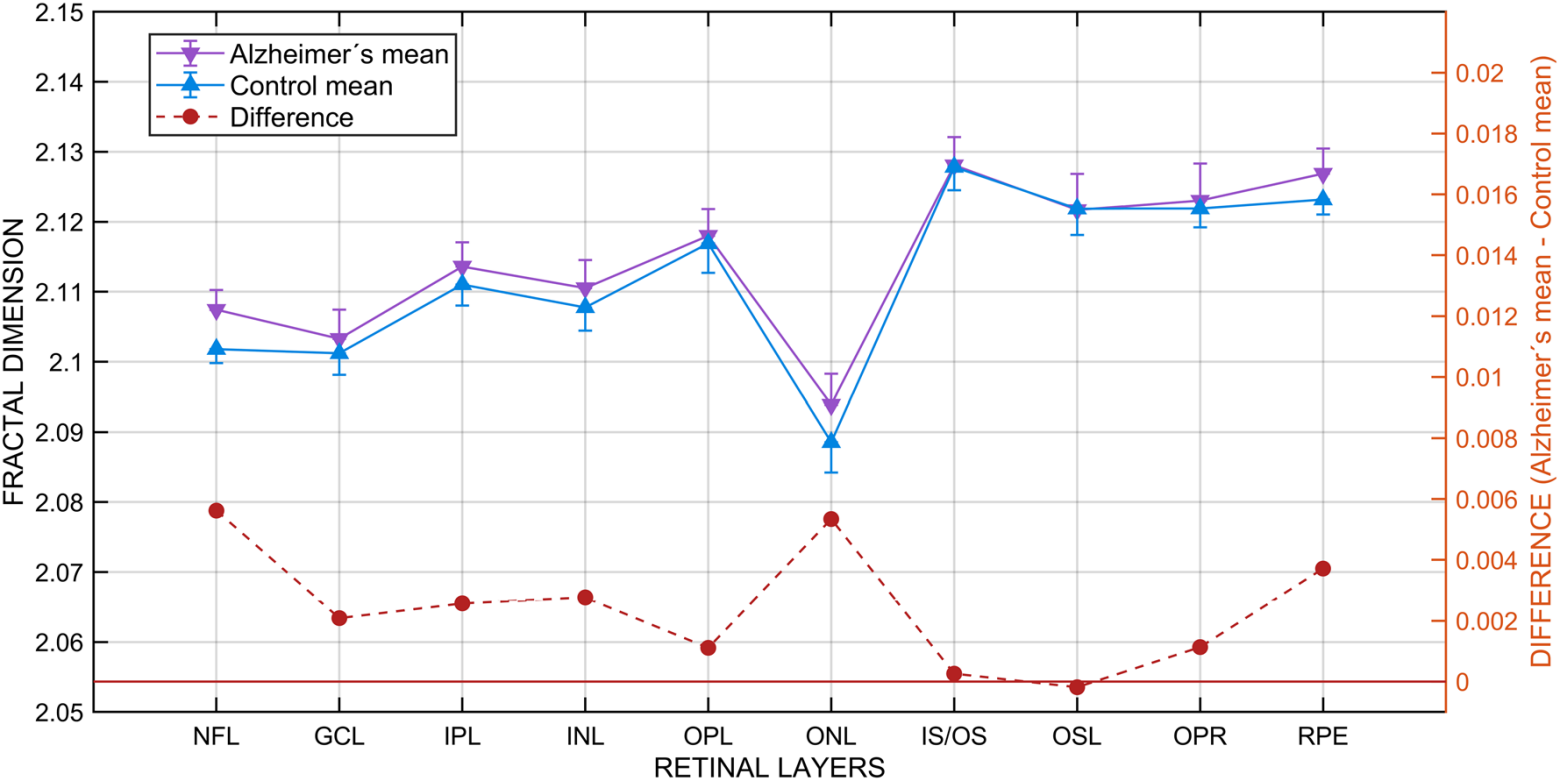
Fractal dimension of retinal layers. Means and standard deviations for each retinal layer in the Alzheimer’s disease and control groups (left axis and continuous lines) and differences between groups (right axis and dashed line). *NFL* nerve fiber layer, *GCL* ganglion cell layer, *IPL* inner plexiform layer, *INL* inner nuclear layer, *OPL* outer plexiform layer, *ONL* outer nuclear layer, *IS/OS* inner segment/outer segment layer, *OSL* outer segment layer, *OPR* outer segment PR/RPE complex, *RPE* retinal pigment epithelium.

Figure 3 gives a graphical representation of Table 2 data and visually demonstrates that the mean FD of most of the layers was higher for the AD group. The comparison of the two paired series of FD confirms that the FD of the AD layers was greater, a difference that was statistically significant (Student’s test for paired samples t = 3.887, df = 9, p = 0.002; Shapiro–Wilk test for normality W = 0.954 with p = 0.77 in the AD group and W = 0.938 with p = 0.57 in the control group; Fisher’s test for homoscedasticity: F = 0.79, df_1_ = 9, df_2_ = 9, p = 0.73). If the comparison is restricted to just the 9 neural layers (excluding the pigment epithelium), the difference is again statistically significant, leading to an identical conclusion (Student’s test for paired samples t = 3.361, df = 8, p = 0.005; Shapiro–Wilk test for normality W = 0.977 with p = 0.99 in patients and W = 0.9621 with p = 0.89 in the controls; Fisher’s test for homoscedasticity: F = 0.74, df_1_ = 8, df_2_ = 8, p = 0.68).

Searching for further information on the effects of AD on each individual layer, we compared the layer’s mean FD in the two independent groups using the Mann–Whitney U statistic, given that most layers did not fit the homoscedasticity and normal distribution requirements for parametric tests. The results of the U statistic and the corresponding p-values are shown in the last two columns of Table 2 and lead to the conclusion that AD has the effect of increasing the FD of most layers, a difference that achieves statistical significance for NFL, ONL and RPE. In contrast, the FD of the total retina was greater in the healthy group, although the difference was not statistically significant.

### Relationship of retinal roughness to AD-related variables

The finding that retinal roughness was greater in the AD group led us to investigate its relationship to other AD-related variables and the prediction of a positive correlation between FD and age and a negative correlation between FD and visual acuity and MMSE. We therefore studied these predictions in all participants and in the AD group separately, the results of which are presented in Table 3 (in italics). The signs of the correlations reaching statistical significance (in italics and boldface) have confirmed all of the above predictions in both samples.

**Table 3:** Roughness correlations.

	All subjects													
	NFL	GCL	IPL	INL	OPL	ONL	IS/OS	OSL	OPR	RPE	Total retina	MMSE	Visual acuity	Age
NFL		**0.43**	**0.49**	**0.43**	0.24	**0.50**	**0.40**	0.19	0.19	**0.41**	0.25	***−0.35***	***−0.45***	***0.38***
GCL	**0.42**		**0.38**	**0.54**	**0.49**	**0.49**	0.17	0.16	0.11	**0.32**	**0.35**	*0.05*	***−0.26***	***0.26***
IPL	**0.59**	**0.41**		**0.74**	**0.54**	**0.52**	**0.61**	**0.35**	**0.26**	**0.59**	**0.31**	*-0.05*	*-0.19*	***0.28***
INL	**0.49**	**0.62**	**0.74**		**0.48**	**0.40**	**0.41**	0.15	0.12	**0.56**	**0.55**	*-0.07*	*-0.18*	***0.26***
OPL	0.32	**0.52**	**0.60**	**0.47**		**0.61**	**0.34**	**0.29**	**0.37**	**0.26**	0.07	*0.08*	*0.07*	*0.02*
ONL	**0.48**	**0.49**	**0.69**	0.34	**0.58**		**0.30**	**0.32**	**0.43**	**0.43**	-0.03	*0.00*	***−0.29***	*0.13*
IS/OS	**0.63**	0.09	**0.67**	**0.39**	0.18	**0.47**		**0.29**	**0.29**	**0.62**	**0.31**	*0.02*	*-0.24*	*0.16*
OSL	0.24	0.14	0.28	0.06	0.30	**0.42**	0.13		**0.75**	0.17	-0.08	*0.14*	*0.08*	*0.13*
OPR	0.27	0.13	0.26	0.03	0.32	**0.41**	0.24	**0.89**		0.22	-0.02	*0.12*	*-0.04*	*0.01*
RPE	**0.41**	0.35	**0.58**	**0.61**	0.16	0.36	**0.66**	***−***0.12	0.03		**0.40**	*-0.18*	***−0.35***	*0.23*
Total retina	**0.58**	**0.45**	**0.51**	**0.76**	0.07	0.04	0.32	***−***0.09	-0.08	**0.56**		0.01	0.03	0.17
MMSE	*0.08*	*0.34*	*0.18*	*0.13*	*0.34*	*0.30*	*0.17*	*0.27*	*0.37*	*0.04*	-0.04		**0.34**	***−*0.43**
Visual acuity	***−****0.37*	*-0.34*	*-0.28*	*-0.17*	*0.10*	***−0.45***	***−0.54***	*-0.01*	***−****0.12*	***−0.57***	-0.13	-0.03		***−*0.32**
Age	*0.10*	*0.22*	*0.26*	*0.29*	*0.00*	*0.04*	***−****0.03*	*0.24*	*0.13*	*0.01*	0.37	0.12	0.09	
	Alzheimer’s disease group													

Mutual correlations of layer roughness and Alzheimer’s disease-related variables for the whole sample (upper triangular matrix) and for the Alzheimer’s disease group (lower triangular matrix). Statistically significant values appear in boldface or bold italics (p < 0.05). Correlations of the 10 retinal layers with non-retinal variables appear in italics.

*NFL*  nerve fiber layer, *GCL* ganglion cell layer, *IPL* inner plexiform layer, *INL* inner nuclear layer, *OPL* outer plexiform layer, *ONL* outer nuclear layer, *IS/OS*  inner segment/outer segment layer, *OSL* outer segment layer, *OPR*  outer segment PR/RPE complex, *RPE *retinal pigment epithelium, *MMSE* Mini-Mental State Examination.

### Two-factor structure of retinal roughness

In view of the relevance and complexity of the relationships among the FDs of the 10 retinal layers, the question arises as to how many independent factors could explain the relationships. A principal component analysis indicated that just 2 factors could account for 50.22% of the total variance. Seeking a deeper understanding of the nature of the 2 factors, we performed a factor analysis on the patient sample and on the entire sample, which in both cases provided almost identical results.

**Figure 4:**
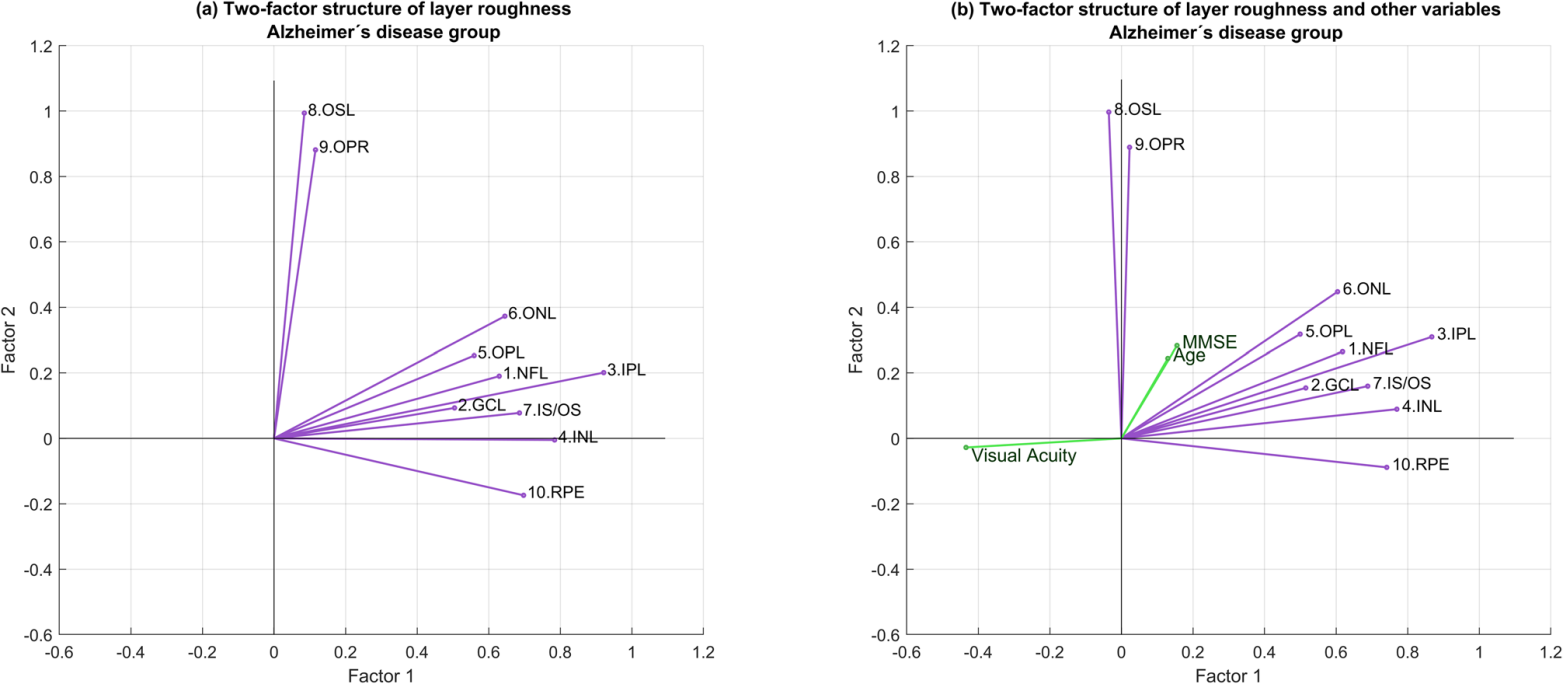
Two-factor structure of retinal roughness. Each vector represents the fractal dimension of a retinal layer; the lower the angle between two vectors, the stronger the correlation between the fractal dimensions of the corresponding layers. **(a)** The two nearly perpendicular bouquets of vectors suggest that layer roughness might be affected by two independent factors -represented by the two coordinate axes- each one acting mainly on the layers corresponding to its bouquet. **(b)** When MMSE, visual acuity and age are included in the factor analysis, only visual acuity shows a relevant and negative relationship with the factor linked to the vector subset headed by inner plexiform layer. The data led to the identification of the two independent factors as the beginning of the retinal inflammatory process (factor 1) and the dysregulation of the choroidal vascular network (factor 2). *NFL* nerve fiber layer, *GCL*  ganglion cell layer, *IPL* inner plexiform layer, *INL* inner nuclear layer, *OPL* outer plexiform layer, *ONL* outer nuclear layer, *IS/OS* inner segment/outer segment layer, *OSL* outer segment layer, *OPR* outer segment PR/RPE complex, *RPE* retinal pigment epithelium, *MMSE* Mini-Mental State Examination.

On the patient sample, the factor analysis confirmed the existence of two factors, unveiled their mutual independence (as implied by factor orthogonality) and confirmed that two factors are sufficient: factor #1 could be linked to the roughness of the IPL together with the NFL, GCL, OPL, ONL, IS/OS and RPE, while factor #2 appears strongly linked to the roughness of the two photoreceptor-related layers: the OSL and OPR (Fig. 4a). When participant’s scores in MMSE, visual acuity and age are included in the analysis (Fig. 4b), only visual acuity showed a strong and negative relationship with factor #1 (for retinal layers only two factors are sufficient: ${\upchi }^{2}$= 30.59, df = 26, p = 0.24; KMO index = 0.65; Bartlett test with ${\upchi }^{2}$= 110.97, df = 45, p < 0.001; det(correlation matrix_10x10_) = 0.0003; when AD related variables added two factors continue to be sufficient: ${\upchi }^{2}$ = 50.7, df = 53 , p = 0.56; KMO index = 0.62; Bartlett test with ${\upchi }^{2}$= 132.8, df = 78, p < 0.001; det(correlation matrix_13x13_) = 0.00003).

To verify the stability of the metric and the results obtained, all the above analyses were repeated with a different numerical thickness coding and a different FD algorithm. In all cases the results have been completely parallel to those just reported.

## Discussion

Roughness needs to be conceptually differentiated from thinning or thickening. Thinning and thickening are local features linked to specific regions of a given layer and can simultaneously adopt different values at different regions of the same layer. In contrast, the roughness of a retinal layer is a global feature whose value is unique to that layer and summarizes the topographical structure of its entire surface. Certainly, roughness might be affected by the intensity and spatial distribution of thinning and thickening processes occurring simultaneously on a retinal layer, but a retinal layer can also undergo global thinning or thickening processes without its roughness being affected. Roughness can increase or decrease, leaving the layer’s mean thickness at any given region of interest unchanged. The roughness of a thickness map is not affected by the smooth undulations that a retinal layer might present in the OCT, resulting from anatomical reality or scan artifacts. When a layer undulates like a flag, its thickness remains unchanged because the thickness must be measured everywhere in the orthogonal direction.

Our results have shown that retinal roughness (assessed by the mean FD of the thickness maps of its layers) is greater in patients with AD than in healthy individuals, a difference that is statistically significant. In our sample, the mean roughness of nine retinal layers in the AD group were greater than that of the controls, and the difference achieved statistical significance in the NFL, ONL and RPE.

With aging, various changes occur in the retinal layers, the most severe of which occur in the RPE, ONL and NFL. RPE cells increase the degree of pleomorphism, with changes in the size and shape of the cells^18^. In ONL, there is a loss of photoreceptors and a displacement of cells towards the IPL, possibly due to changes in the shape of the cones with aging^19–21^. In addition, there is an activation of glial cells, astrocytes, Müller cells^22^ and microglia^23^. In NFL, the axonal diameters show changes with age, with some axons showing a well-preserved internal structure and other axons swelling with accumulations of abnormal organelles^24,25^. All of these changes, which could be accentuated in patients with AD, can help explain (along with atrophy and inflammatory processes) the statistically significant increase in the roughness of these 3 retinal layers. The results of the study by Song et al.^26^ are consistent with this explanation. The authors investigated the structural homogeneity of the tissue forming the NFL, OPL and RPE layers in the retinas of triple transgenic AD mice and found a statistically significant higher correlation slope. The authors’ results indicate higher tissue heterogeneity in those layers of AD mice, which, although resulting from mouse AD models, indicate that the increased roughness found on the thickness maps of human AD retinal layers could be explained by the abovementioned structural changes in their tissue formation.

The FD of the entire retina was slightly greater in the controls than in the patients. The difference is therefore in direct contrast to that found for most of the retina’s component layers. At first glance, this result might be perceived as contradicting the hypothesis that roughness is greater in AD; however, this result could have been anticipated, because the thickness of the entire retina roughly results from adding the thickness of all of its layers. When thickened regions of different layers overlap, an increase in roughness of the total retina should be expected. The results of the study by Jáñez-Escalada et al.^16^ have however shown that in patients with AD thickened regions of different layers tend to appear in different retinal positions, implying smoothing of the delimiting surfaces of the entire retina and decreasing its roughness, which we found in our study.

The correlations between NFL FD and cognitive impairment, visual acuity and age reinforce the link between retinal roughness and the development of AD and serve as an invitation to investigate the use of roughness in the follow-up of AD. The negative correlations between roughness and visual acuity found in the NFL, GCL, ONL and IS/OS are consistent with the decreased contrast sensitivity to high spatial frequency found by Salobrar-García et al.^6^

The results from factor analysis show that two independent factors affect the various retina layers. Factor 1 is intrinsically related to IPL, NFL, GCL, IS/OS and INL, which correspond mostly to the innermost layers of the retina. Previous studies have observed that these layers contain a greater number of amyloid β deposits^27–33^, leading to an inflammatory process, with activation of retinal glia, prior to the neurodegenerative process^34^. In experimental studies, microglia activation and migration in the layer and between retinal layers have been observed in 3xTg-AD mice^35^. Therefore, the factor 1 that affects these layers could be the start of the retinal inflammatory process.

Factor 2 correlates more closely with the OSL and OPR, layers located on the outer retina and related to the choroid. Choroidal vascularization, which is essential for the outermost layers of the retina and macula, is affected even in these very early stages of AD, resulting in a thinning of the choroid, with no involvement of the retinal vascular network^12^. These two vascular networks differ, and their flow in the choroid is regulated by neurons^36–38^. Therefore, the factor 2 could be identified as the starting dysregulation of the choroidal vascular network.

Of the 2635 reviewed patients with AD, only 19 were ultimately included in the study’s AD group. The small number of patients in our AD group is the result of a deliberate strategy to obtain a highly homogeneous patient group, all of whom are in a very similar stage of AD development. Another possible approach would have been to include more patients with greater variability by relaxing the inclusion criteria. Although this strategy is more frequently adopted (when the choice is feasible), our findings of statistically significant differences in FD between the patient and control groups have shown the usefulness of the strategy adopted in present study.

The surface roughness of a retinal layer can be directly observed and assessed on its two delimiting surfaces; however, this approach has a relevant drawback: the lack of independence of measurements from different layers due to their physical contact or proximity. The delimiting surface of any given layer is shared by the adjacent layer; therefore, a thickened region caused by AD in a layer will push into the contacting region of the adjacent layer, thereby creating a spurious roughness in the bounding surfaces of the adjacent layer which does not correspond to its internal structure. Extending the same argument to other layers, we can conclude that the curvature produced by a single thickened region of a single layer may curve the surfaces of all other layers. Therefore, investigating the roughness of delimiting surfaces would render impossible to distinguish which layer is the one affected by AD. The solution to this problem is to mathematically flatten the outer delimiting surface of each layer, so that its thickening and thinning will manifest only in curvature changes of the opposite delimiting surface. But this new curved surface -associating to each retinal point the thickness of the layer at that point- is exactly coincident with the thickness map of the retinal layer. This is the main reason to focus our analysis on the thickness maps of retinal layers, instead of their delimiting surfaces. Another reason comes from the vertical misalignment of the B-cans, which could represent a powerful source of noise directly affecting roughness measurements on layer surfaces. Therefore, our study focused only on the roughness of layer thickness because it contains the same information as the delimiting surfaces, it allows identifying the retinal layer suffering thickness changes and is less sensitive to the noise raised by technical artifacts.

The selection of the appropriate roughness index proved to be complex due to the large number of possibilities available. Roughness has attracted the interest of researchers in the industry and scientific sectors (regarding the quality of polished metallic surfaces, terrain surface description, surfaces of rock fractures, etc.) and a large number of indexes have been defined and employed, such as the standard deviation of elevations, slopes, curvatures, ratios of surface to scanned areas, gradient modules and orientations, directional slopes, etc^39^**.** However, the noise inherent to OCT imaging may strongly affect this type of index and thwarted our initial approaches to quantify roughness through the statistics of local features. We therefore employed a different approach based on fractal geometry^40,41^: to assess the roughness of a retinal layer through the fractal dimension of its thickness map. FD is a mathematical measure of complexity that has previously been employed in the retinal research of blood vessels^42,43^ and diagnosis of diabetic retinopathy^44^.

Our study had a number of limitations. The algorithm to calculate the FD, despite its positive evaluation, still has some drawbacks that need to be addressed^45^. The scanned retinal area was 6 × 6 mm^2^; however, our analysis had to be restricted to a square measuring only 2.555 × 2.555 mm^2^ due to practical constraints whose future removal might allow for improvements. Lastly, FD stability might benefit from spatial isotropy of the scanned area, a feature lacking in foveally centered regions, which are strongly anisotropic, an observation that warrants study to search for roughness differences in retinal regions not centered on the fovea or on the optic nerve head.

Certain characteristics might render our results useful in clinical practice. The roughness of retinal layers captures their topographical complexity with a single value, is a holistic feature specific for each retinal layer, can be calculated in less than a second, integrates the information coming from thinned and thickened regions on the same layer, and is a potentially useful biomarker for Alzheimer’s disease at a very early stage of the disease. Jointly, these characteristics pave the way for using FD to diagnose and follow-up individuals with AD.

In retinal analysis, roughness can be viewed as a new dimension emerging from the long-lasting controversies regarding the thinning and thickening of retinal layers in AD research. Roughness integrates the two processes and quantifies their combination at different spatial scales. The surface roughness of retinal layers and its quantification through the FD of their thickness maps represent two innovations in the conceptual and methodological fields, respectively. The results of their application in AD research open the door to exploring their usefulness in the research of other neurodegenerative diseases whose effects on the central nervous system also have observable correlates in the retina.

## Methods

### Participants

The current proposal that the roughness of retinal layers -quantified by the fractal dimension of their thickness maps- is a biomarker of AD has been tested with the data set of our previous publication^16^, which was intended to locate thinned and thickened retinal regions using random field theory; it also provided the detailed description of participants and OCT imaging that we summarize below.

This cross-sectional study^16^ recruited patients with AD from the Memory Unit of the Geriatric Service of the Clinic Hospital San Carlos (Madrid, Spain). The study protocol followed the principles of the Declaration of Helsinki and was approved by the Institutional Ethics Committee for Clinical Research of Clinic Hospital San Carlos (code number 11/372-E). All participants gave their written informed consent.

We reviewed 2635 patient records to identify 87 patients with mild AD, defined as GDS 4 according to the NINCDS-ADRDA Alzheimer's Criteria^16^. These patients underwent a full neurological examination and brain magnetic resonance imaging to rule out alternative diagnoses. Those patients previously diagnosed with an ophthalmological disease (glaucoma or suspected glaucoma, media opacity, and retinal diseases) were excluded. The remaining 29 patients with mild AD and free of ocular disease and systemic disorders that might affect their vision, along with 37 age-matched healthy control participants who scored above 27 on the Mini-Mental State Examination (MMSE) underwent a complete ophthalmological examination. We subsequently excluded 6 patients and 9 controls due to posterior pole conditions including drusen, macular degeneration, suspicion of glaucoma, glaucoma, epiretinal membrane and cataracts. We then excluded 4 patients and 3 controls due to inconsistent signal intensity across the OCT scan. The remaining 19 patients and 25 controls passed a complete ophthalmologic examination conducted by the same clinician, including an assessment of visual acuity and refraction, a slit-lamp analysis of the anterior and posterior segments of the eye, applanation tonometry (Perkins MKII tonometer, Haag-Streit Reliance Medical, Switzerland), dilated fundus examination and OCT. All participants showed an AREDS Clinical Lens Standards grade < 2, a best-corrected visual acuity of 20/40, spherocylindrical refraction within ± 5 diopters and an intraocular pressure < 20 mm Hg. Another participant in the control group was excluded after enrollment because automatic segmentation of his retinal layers became unfeasible. Ultimately, the study included 19 patients and 24 controls. Only the right eye of each participant was studied, except in 4 patients whose left eye was studied instead, because data for their right eye did not reach the necessary level of quality. These patients’ OCT data were left–right flipped so that the temporal-nasal anatomical areas were matched for all participants.

### OCT imaging

We obtained optical coherence volumes after pupil dilatation using a spectral domain OCT (3D OCT-1000 Topcon, Japan) ^16^. We obtained 3 high-quality peripapillary and macular images in a raster pattern covering a 6 × 6 mm^2^ area with a scan density of 512 × 128 pixels in approximately 2.5 s (27,000 A scans/sec). The voxel size was 11.7 × 46.9 × 3.5 µm^3^ (horizontal x vertical x depth), according to the calibration provided by the manufacturer. All OCT images were acquired by the same experienced technician, with the light beam entry point always centered on the pupil to avoid oblique scanning artifacts. Images were reviewed for quality, and the criteria for acceptable fundus images were as follows: (a) no large eye movements, defined as an abrupt shift completely disconnecting a large retinal vessel; (b) consistent signal intensity across the scan; and (c) no black bands (caused by blinking) throughout the examination. In addition, the criteria for acceptable scanning were a signal-to-noise ratio > 30 and an A-scan acceptance > 95% during fast NFL scanning. Therefore, an OCT was obtained from 24 healthy subjects and 19 AD patients for a 6 × 6 mm^2^ foveally centered retinal square.

### Analysis steps in OCT postprocessing

**Figure 5:**
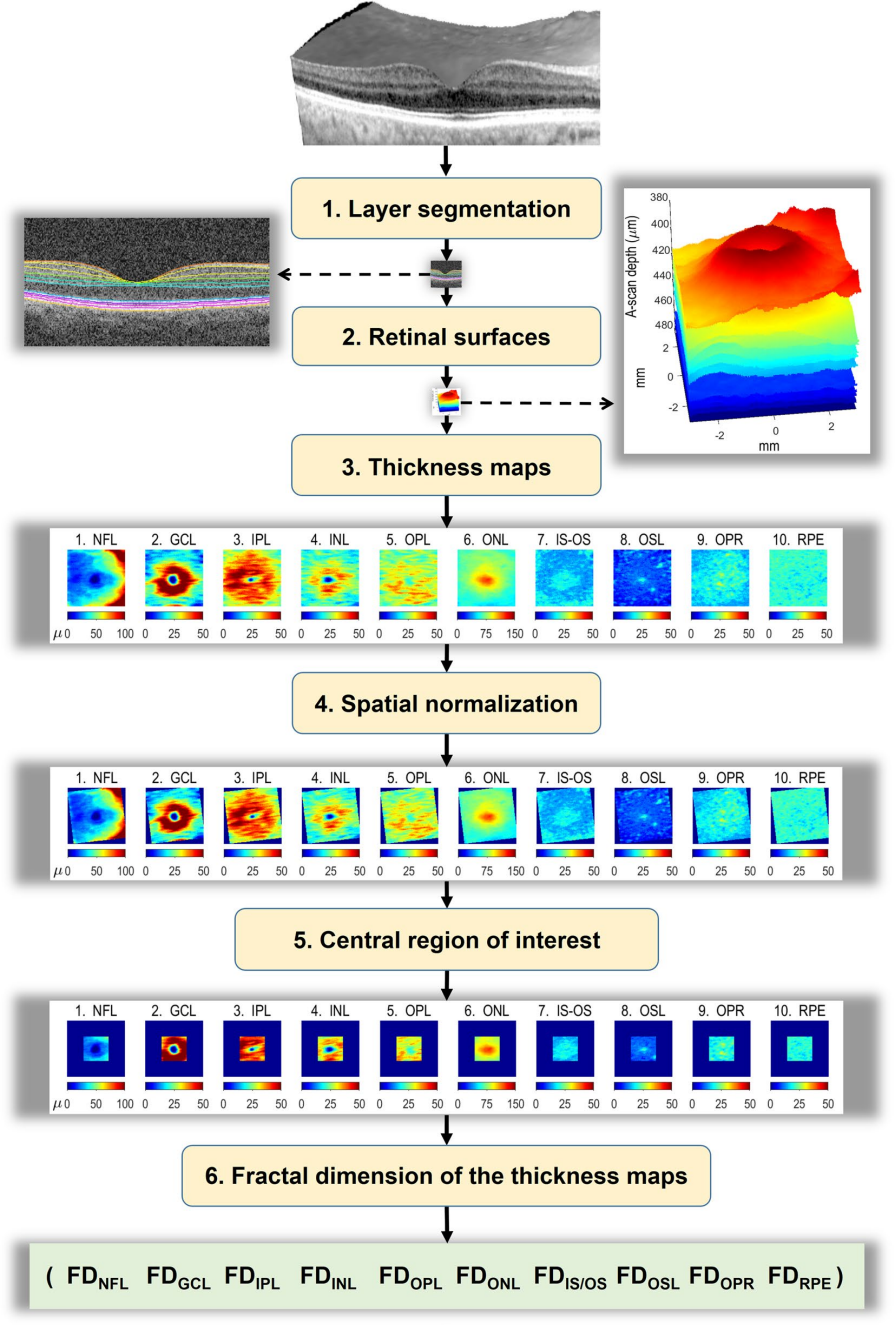
OCT postprocessing. (1)* Layer segmentation.* In each OCT, the 10 retinal layers listed below were separated using a fully automatic algorithm. (2) Retinal surfaces. We obtained the 11 surfaces delimiting the 10 retinal layers listed below. (3) Thickness maps. At each retinal point scanned, the thickness of the retinal layer was calculated as the distance between its two bounding surfaces in the direction orthogonal to the layer. (4) Spatial normalization. The set of the 10 thickness maps of each participant was moved, rotated and scaled so that the macular and papillar centers of all subjects overlapped (5) Central region of interest. The largest square region available in all layers for all subjects was selected for analysis, its side being 2.555 mm. (6) Fractal dimension of the thickness map in the central square was finally calculated as the index of its roughness. *FD* fractal dimension, *NFL* nerve fiber layer, *GCL* ganglion cell layer, *IPL* inner plexiform layer, *INL* inner nuclear layer, *OPL* outer plexiform layer, *ONL* outer nuclear layer, *IS/OS* inner segment/outer segment layer, *OSL* outer segment layer, *OPR* outer segment PR/RPE complex, *RPE* retinal pigment epithelium. Images created using MATLAB (2018a) www.mathworks.com/products/matlab, Layer Segmentation Module of The Iowa Reference Algorithms (3.6) www.iibi.uiowa.edu/oct-reference and Heidelberg Eye Explorer (1.10.4.0) www.HeidelbergEngineering.com.

The following steps, summarized in Fig. 5, were applied to the OCTs from all participants.

1. Layer segmentation. We exported raw data from the macular and papillary spectral domain OCT (or, in some cases, from their DICOM version) and segmented the 10 layers using the Layer Segmentation Module (Iowa Reference Algorithms 3.6 Retinal Image Analysis Lab, Iowa Institute for Biomedical Imaging, Iowa City, IA, USA)^46–48^.
2. Retinal surfaces. We decoded the resulting *xml* files with an in-house MATLAB program to obtain the 3D coordinates of the 11 bounding surfaces shown in Fig. 6, the macular and papillary centers, and the masks of the regions where automatic segmentation failed. In the subsequent analyses we ignored all data from the A-scans in the regions where the segmentation failed.

**Figure 6:**
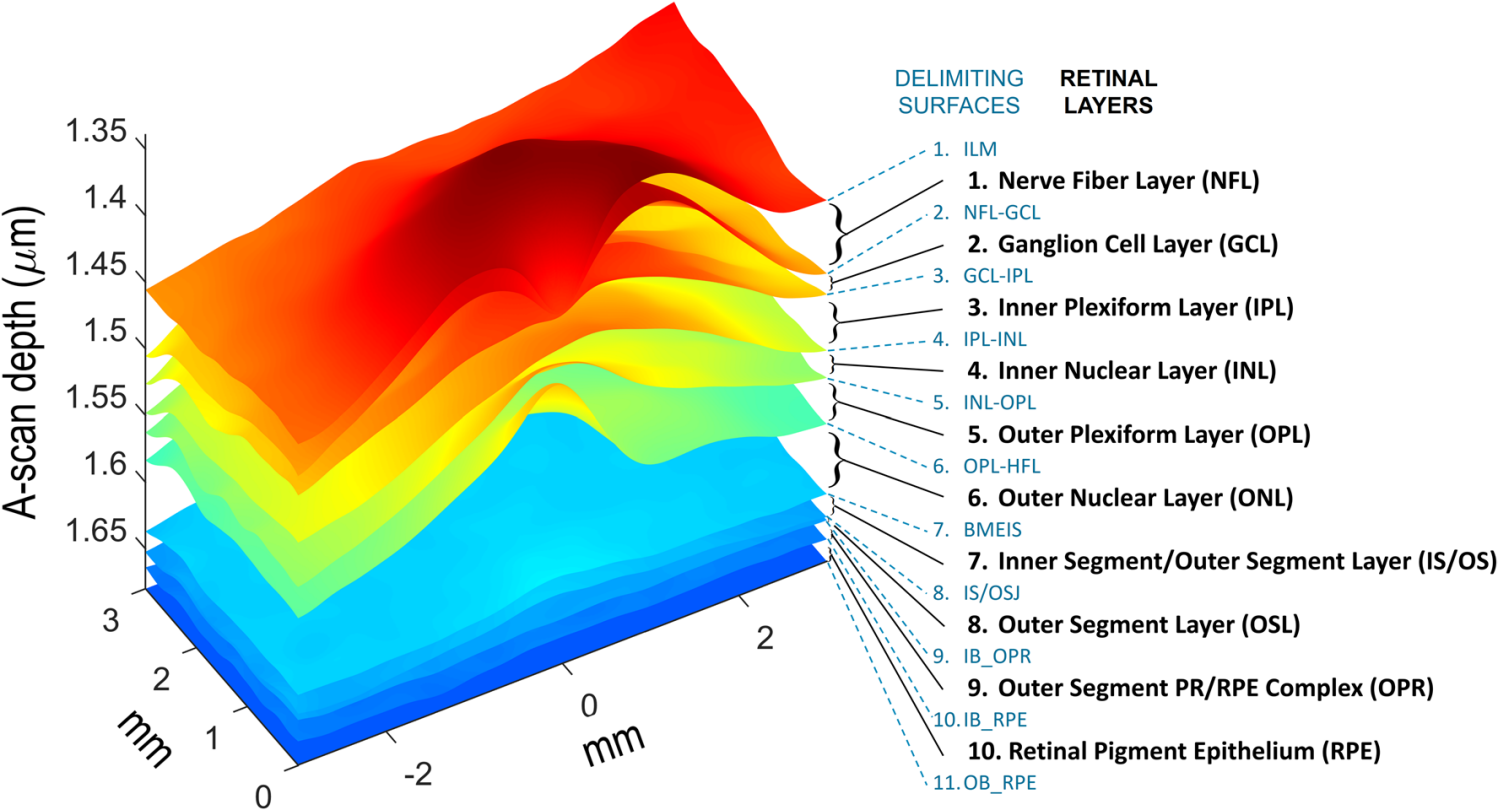
Retinal layers and their delimiting surfaces. Retinal layers: (1) nerve fiber layer (NFL), (2) ganglion cell layer (GCL), (3) inner plexiform layer (IPL), (4) inner nuclear layer (INL), (5) outer plexiform layer (OPL), (6) outer nuclear layer (ONL), (7) inner segment/outer segment layer (IS/OS), (8) outer segment layer (OSL), (9) outer segment PR/RPE complex (OPR), (10) retinal pigment epithelium layer (RPE). Delimiting surfaces: (1) inner limiting membrane (ILM), (2) nerve fiber layer-ganglion cell layer (NFL-GCL), (3) ganglion cell layer-inner plexiform layer (GCL-IPL), (4) inner plexiform layer-inner nuclear layer (IPL-INL), (5) inner nuclear layer-outer plexiform layer (INL-OPL), (6) outer plexiform layer-Henle fiber layer (OPL-HFL), (7) boundary of myoid and ellipsoid of inner segments (BMEIS), (8) inner segment-outer segment junction (IS/OSJ), (9) inner boundary of OPR (IB_OPR; OPR: outer segment PR/RPE complex), (10) inner boundary of retinal pigment epithelium (IB_RPE), and (11) outer boundary of retinal pigment epithelium (OB_RPE). Image created using MATLAB (2018a) www.mathworks.com/products/matlab.
3. Thickness maps. The raw thickness of the retinal layer was calculated at each scanned point as the distance between the two bounding surfaces of the layer; but this method tends to overestimate the true thickness because the distance between the two surfaces is measured in the direction of the A-scan, which usually departs a certain angle from the direction perpendicular to the layer; therefore, that angle was calculated and the true thickness was obtained as the product of its cosine times the raw thickness.
4. Spatial normalization. Although the OCT scanning protocol was the same for all participants, interpersonal differences in the size and shape of the eyeball imply that the anatomical region actually scanned varies from subject to subject. To ensure that the same functional and anatomical regions are studied in the retinas of all of them, the thickness maps of each participant were moved, rotated and scaled to place the foveal fossa in the center of the image and to render the maculopapillary axis 4.377 mm long and 6.766° tilted, which are the mean values in our sample. A more detailed description of steps 1 to 4 can be found in a previous article^16^.
5. Central region of interest. After the processing described above, the retinal area left for analysis was not the same in all subjects, mainly because automatic segmentation failed in a few peripheral regions and because OCT rotation and scaling during spatial normalization yielded peripheral regions with no data. Thus, the roughness analysis had to be restricted to the largest square region -centered on the fovea- whose normalized thickness map was available from all participants: a square 2.555 mm on a side.
6. Fractal dimension of the thickness maps**.** In the central square of each thickness map we calculated the FD as the index of its roughness: FD captures the roughness of each layer in just one number slightly higher than 2. The *box counting* method was considered the most appropriate method for estimating FD because retinal layers are not strictly self-similar. Given the mathematical equivalence of the thickness map and its image representation, we selected the algorithm known as *integer ratio differential box counting* for grayscale images^49^ due to its suitable properties compared to a wide set of alternative algorithms^39^. The computer program that implemented this algorithm was written in MATLAB R2018a and run on an Intel Xeon processor E5-2690 v3 (12 core, 2.6 GHz, 35 MB, and 32 GB RAM).

### Statistical analysis

We compared the FDs of the retinal layers between the patients and controls. The dependent variable FD is dimensionless, continuous and quantitative. We employed Student’s t-test for independent samples to evaluate the FD differences between the AD and control groups. We applied its version for paired samples to compare the differences between the diseased and healthy layers and a mixed design analysis of variance was planned to determine the effects and interaction on FD of the groups and layers if homoscedasticity and normality were satisfied. We employed alternative non-parametric tests when the data did not fit the requirements of the parametric tests. All variables of interest are quantitative. We therefore studied their relationships using Pearson correlations and dimensionality reduction techniques: principal component and factor analysis. Level for alpha error was 0.05. One-sided tests were used to test directional hypotheses on mean differences and correlations. All statistical tests were conducted twice, using standard routines available in MATLAB and R, and the equivalence of the results was checked in all cases.

### Ethics approval and consent to participate

The study protocol followed the principles of the Declaration of Helsinki and was approved by the Ethics Committee for Clinical Research of the Hospital Clínico San Carlos (code number 11/372-E). All participants gave their written informed consent.

## Data Availability

The retinal thickness dataset analyzed in the current study is available from the Figshare repository at https://dx.doi.org/10.6084/m9.figshare.8323334.

## References

[1] Sadun AA, Borchert M, DeVita E, Hinton DR, Bassi CJ (1987). Assessment of visual impairment in patients with Alzheimer’s disease. Am. J. Ophthalmol..

[2] Mendez MF, Tomsak RL, Remler B (1990). Disorders of the visual system in Alzheimer’s disease. J. Clin. Neuroophthalmol..

[3] Cronin-Golomb A, Rizzo JF, Corkin S, Growdon JH (1991). Visual function in Alzheimer’s disease and normal aging. Ann. N. Y. Acad. Sci..

[4] Polo V (2017). Visual dysfunction and its correlation with retinal changes in patients with Alzheimer’s disease. Eye (Lond)..

[5] Salobrar-Garcia E (2015). Ophthalmologic psychophysical tests support OCT findings in mild Alzheimer’s disease. J. Ophthalmol..

[6] Salobrar-García E (2019). Changes in visual function and retinal structure in the progression of Alzheimer’s disease. PLoS One.

[7] Shen Y (2013). The attenuation of retinal nerve fiber layer thickness and cognitive deterioration. Front. Cell. Neurosci..

[8] Oktem EO (2015). The relationship between the degree of cognitive impairment and retinal nerve fiber layer thickness. Neurol. Sci..

[9] Salobrar-Garcia E (2015). Analysis of retinal peripapillary segmentation in early Alzheimer’s disease patients. Biomed Res. Int.

[10] Cunha LP (2016). Macular thickness measurements with frequency domain-OCT for quantification of retinal neural loss and its correlation with cognitive impairment in Alzheimer’s disease. PLoS One..

[11] Trebbastoni A (2016). Retinal nerve fibre layer thickness changes in Alzheimer’s disease: Results from a 12-month prospective case series. Neurosci. Lett..

[12] Salobrar-Garcia E (2020). Ocular vascular changes in mild Alzheimer’s disease patients: Foveal avascular zone, choroidal thickness, and ONH hemoglobin analysis. J. Pers. Med..

[13] Choi SH, Park SJ, Kim NR (2016). Macular ganglion cell-inner plexiform layer thickness is associated with clinical progression in mild cognitive impairment and Alzheimers disease. PLoS One..

[14] Garcia-Martin E (2016). Ganglion cell layer measurements correlate with disease severity in patients with Alzheimer’s disease. Acta Ophthalmol..

[15] Lad EM (2018). Evaluation of inner retinal layers as biomarkers in mild cognitive impairment to moderate Alzheimer’s disease. PLoS One..

[16] Jáñez-Escalada L (2019). Spatial analysis of thickness changes in ten retinal layers of Alzheimer’s disease patients based on optical coherence tomography. Sci. Rep..

[17] Zawada DG, Hopley D (2011). Reef topographic complexity. Encyclopedia of Modern Coral Reefs: Structure, Form and Process.

[18] Friedman E, Ts’o MOM (1968). The retinal pigment epithelium: II. Histologic changes associated with age. Arch. Ophthalmol..

[19] Gartner S, Henkind P (1981). Aging and degeneration of the human macula. I. Outer nuclear layer and photoreceptors. Br. J. Ophthalmol..

[20] Liem AT, Keunen JE, Van Norren D, Van de Kraats J (1991). Rod densitometry in the aging human eye. Investig. Ophthalmol. Vis. Sci..

[21] Gao H, Hollyfield JG (1992). Aging of the human retina: Differential loss of neurons and retinal pigment epithelial cells. Investig. Ophthalmol. Vis. Sci..

[22] Ramírez JM, Ramírez AI, Salazar JJ, De Hoz R, Trivio A (2001). Changes of astrocytes in retinal ageing and age-related macular degeneration. Exp. Eye Res..

[23] Ramírez AI (2020). Microglial changes in the early aging stage in a healthy retina and an experimental glaucoma model. Prog. Brain Res..

[24] Avendano J, Rodrigues MM, Hackett JJ, Gaskins R (1980). Corpora amylacea of the optic nerve and retina: A form of neuronal degeneration. Investig. Ophthalmol. Vis. Sci..

[25] Zhu Y (2018). Ultrastructural morphology of the optic nerve head in aged and glaucomatous mice. Investig. Ophthalmol. Vis. Sci..

[26] Song G (2020). Multimodal coherent imaging of retinal biomarkers of Alzheimer’s disease in a mouse model. Sci. Rep..

[27] Hardy J, Selkoe DJ (2002). The amyloid hypothesis of Alzheimer’s disease: Progress and problems on the road to therapeutics. Science.

[28] Selkoe DJ (2004). Cell biology of protein misfolding: the examples of Alzheimer’s and Parkinson’s diseases. Nat. Cell Biol..

[29] Selkoe DJ (2008). Soluble oligomers of the amyloid beta-protein impair synaptic plasticity and behavior. Behav. Brain Res..

[30] Alexandrov PN, Pogue A, Bhattacharjee S, Lukiw WJ (2011). Retinal amyloid peptides and complement factor H in transgenic models of Alzheimer’s disease. NeuroReport.

[31] Ratnayaka JA, Serpell LC, Lotery AJ (2015). Dementia of the eye: the role of amyloid beta in retinal degeneration. Eye (Lond)..

[32] Hart NJ, Koronyo Y, Black KL, Koronyo-Hamaoui M (2016). Ocular indicators of Alzheimer’s: exploring disease in the retina. Acta Neuropathol..

[33] Ramirez AI (2017). The role of microglia in retinal neurodegeneration: Alzheimer’s disease, Parkinson, and glaucoma. Front. Aging Neurosci..

[34] Grimaldi A (2018). Inflammation, neurodegeneration and protein aggregation in the retina as ocular biomarkers for Alzheimer’s disease in the 3xTg-AD mouse model. Cell Death Dis..

[35] Salobrar-García E (2020). Microglial activation in the retina of a triple-transgenic Alzheimer’s disease mouse model (3xTg-AD). Int. J. Mol. Sci..

[36] Schrödl F (2003). Intrinsic choroidal neurons in the human eye: Projections, targets, and basic electrophysiological data. Invest. Ophthalmol. Vis. Sci..

[37] Triviño A (2005). NPY and TH innervation in human choroidal whole-mounts. Histol. Histopathol..

[38] de Hoz R (2008). Substance P and calcitonine gene-related peptide intrinsic choroidal neurons in human choroidal whole-mounts. Invest. Ophthalmol. Vis. Sci..

[39] Stout K (2000). Development of Methods for Characterisation of Roughness in Three Dimensions.

[40] Mandelbrot BB (1983). The Fractal Geometry of Nature/Revised and Enlarged Edition.

[41] Pentland AP (1984). Fractal-based description of natural scenes. IEEE Trans. Pattern Anal. Mach. Intell..

[42] Lemmens S (2020). Systematic review on fractal dimension of the retinal vasculature in neurodegeneration and stroke: Assessment of a potential biomarker. Front. Neurosci..

[43] Somfai GM (2014). Fractal-based analysis of optical coherence tomography data to quantify retinal tissue damage. BMC Bioinform..

[44] Gao W (2016). Two-dimensional fractal analysis of retinal tissue of healthy and diabetic eyes with optical coherence tomography. J. Biomed. Photon. Eng.

[45] Panigrahy C, Seal A, Mahato NK, Bhattacharjee D (2019). Differential box counting methods for estimating fractal dimension of gray-scale images: A survey. Chaos, Solitons Fractals.

[46] Garvin MK (2009). Automated 3-D intraretinal layer segmentation of macular spectral-domain optical coherence tomography images. IEEE Trans. Med. Imaging.

[47] Antony B (2011). Automated 3-D method for the correction of axial artifacts in spectral-domain optical coherence tomography images. Biomed. Opt. Express.

[48] Sonka M, Abràmoff MD (2016). Quantitative analysis of retinal OCT. Med. Image Anal..

[49] Long M, Peng F (2013). A box-counting method with adaptable box height for measuring the fractal feature of images. Radioengineering.

